# Carcinoid Tumor Arising from the Sphenoid Sinus Treated with Definitive Intensity-modulated Radiation Therapy: A Case Report

**DOI:** 10.7759/cureus.4288

**Published:** 2019-03-21

**Authors:** Lara Hilal, Mustafa Jammal, Ibrahim Khalifeh, Arafat Tfayli, Bassem Youssef

**Affiliations:** 1 Radiation Oncology, American University of Beirut Medical Center, Beirut, LBN; 2 Pathology, American University of Beirut Medical Center, Beirut, LBN; 3 Oncology, American University of Beirut Medical Center, Beirut, LBN

**Keywords:** carcinoid, neuroendocrine cancer, radiotherapy, paranasal sinuses, treatment, neuroendocrine tumor (net)

## Abstract

Head and neck neuroendocrine tumors (NET) are a rare type of cancer. NET can be classified according to the histopathological features. The typical carcinoid tumor is a well-differentiated tumor that is the least common among other types. Owing to its indolent behavior and variable radiological and pathological features, treatment of carcinoid tumors remains a challenge. We report a case of a 54-year-old man presenting with a non-operable carcinoid tumor arising in the sphenoid sinuses treated with radiotherapy with stable disease control after three years follow-up.

## Introduction

Neuroendocrine tumors (NETs) are a group of neoplasms with various clinical presentations and growth rates. Although rare, neuroendocrine carcinoma has been well-described in head and neck cancers [1]. Of all of the other types of NETs, well-differentiated NET, also known as typical carcinoid, is the least common in the head and neck and occurs most commonly in the supraglottic larynx [2]. Very few cases of typical carcinoid tumor arising from the paranasal sinuses have been reported in the literature. The standard treatment for such tumors has not been yet established, with surgery playing the major role [3]. Herein, we report a case of unresectable carcinoid tumor arising from the sphenoid sinus of a 54-year-old man successfully treated with definitive radiotherapy.

## Case presentation

A 54-year-old man presented in 2015 with a 12-month history of persistent left nasal obstruction and nasal discharge, as well as episodes of self-resolving epistaxis. He was first treated for presumed nasal polyps and sinusitis without improvement. The patient is known to have hypertension. He denied any history of tobacco or alcohol use and had no prior history of radiation.

On physical examination, the patient had a Karnofsky Performance Status (KPS) of 90%. On fiber-optic examination, there was evidence of a mass filling the left nasal cavity. No palpable cervical lymphadenopathy was noted. There was no evidence of neurological deficits, and cranial nerves, II to XII, were intact.

**Figure 1:**
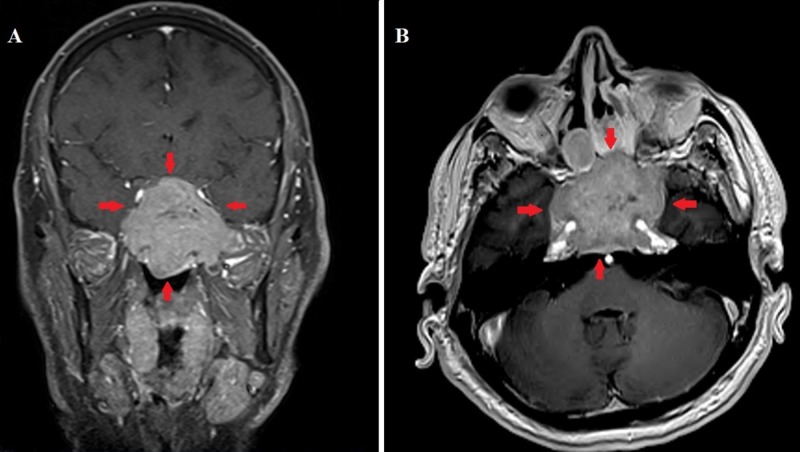
Coronal (A) and axial (B) T1-weighted magnetic resonance images with contrast showing a large unresectable sphenoid sinus tumor.

A computed tomography (CT) scan and magnetic resonance imaging (MRI) of the head and paranasal sinuses revealed a large, enhancing soft tissue mass (6.1 x 4.9 x 4.1 cm) centered in the sphenoid sinus with an invasion of the base of the skull and clivus(Figure 1). There was also the destruction of the greater wings of the sphenoid, more on the left side, with an invasion of the left pterygopalatine fossa and extension into the left masticator space. The mass involved the sellar region and the cavernous sinus, as well as the internal carotid artery canals. The internal carotid arteries were still patent. The mass was extending and invading the posterior aspect of the ethmoid air cells. There was complete opacification of the left nasal cavity and the maxillary sinuses with obliteration of the ostiomeatal complexes by mucosal disease.

**Figure 2:**
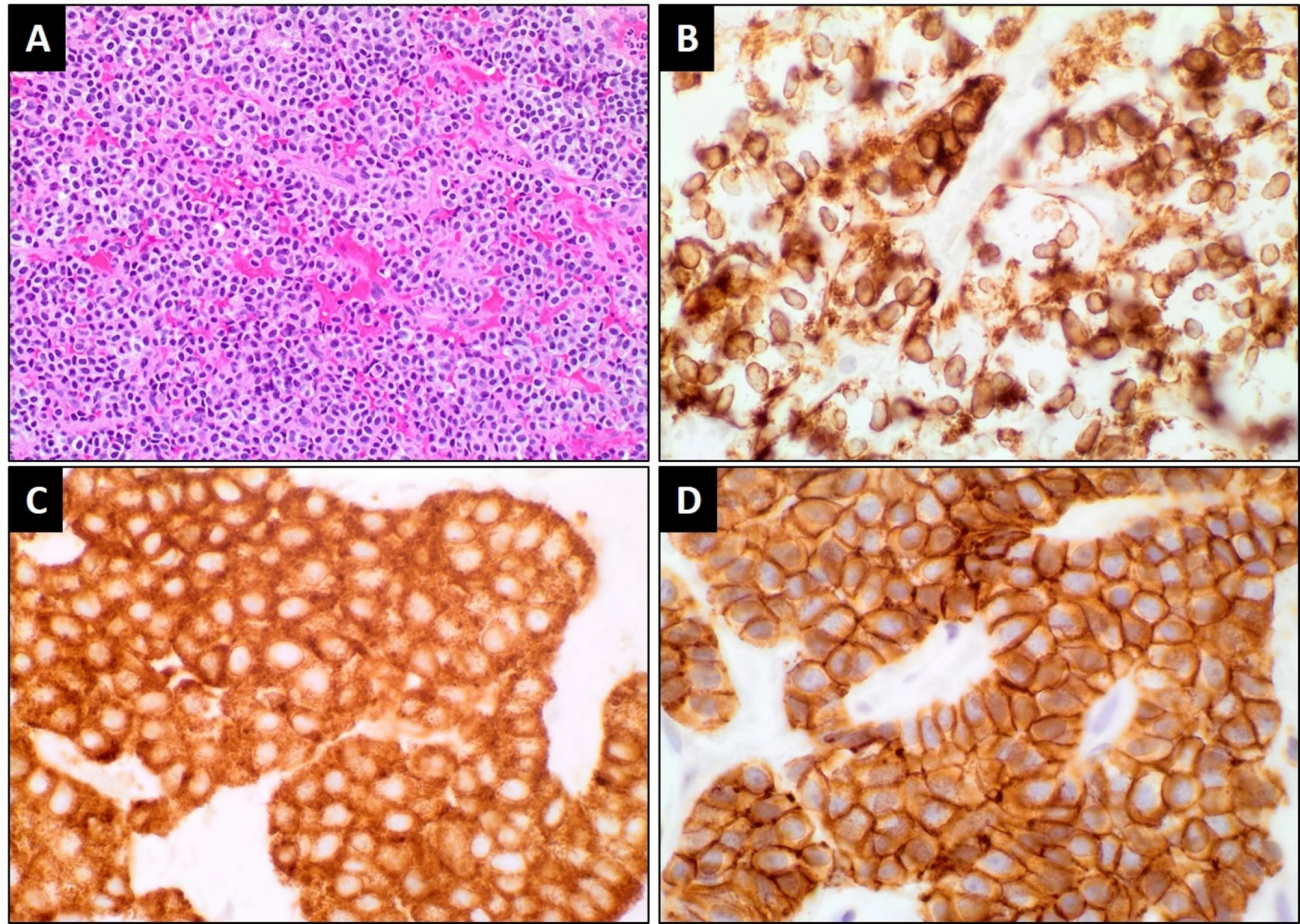
Representative histological sections displaying typical carcinoid tumor (A) Sections show a well-vascularized tumor composed of cords and sheets of monotonous cells with round nuclei and a moderate amount of granular cytoplasm with no brisk mitotic activity or necrosis. The tumor cells stain diffusely for (B) CKAE1/3, (C) synaptophysin, and (D) CD56.

A transnasal biopsy of the lesion was performed. Morphological examination revealed well-vascularized sheets and cords of uniform round cells with a moderate amount of pink, finely granular cytoplasm. Tumor cells were strongly and diffusely positive for synaptophysin (Syn), chromogranin A (CgA), CD56, and cytokeratin AE1/AE3. They were negative for CK7, CK20, and S-100. The Ki-67 proliferative index was less than 1%. The pathological diagnosis was a typical carcinoid neuroendocrine tumor of the sphenoid sinus (Figure 2).

A CT scan of the chest and galium positron emission tomography (PET) scan revealed no evidence of regional or distant metastasis. Clinical staging of this tumor, based on the American Joint Committee on Cancer (AJCC) TNM staging system for the nasal cavity and paranasal sinuses [4], was T4bN0M0. 

**Figure 3:**
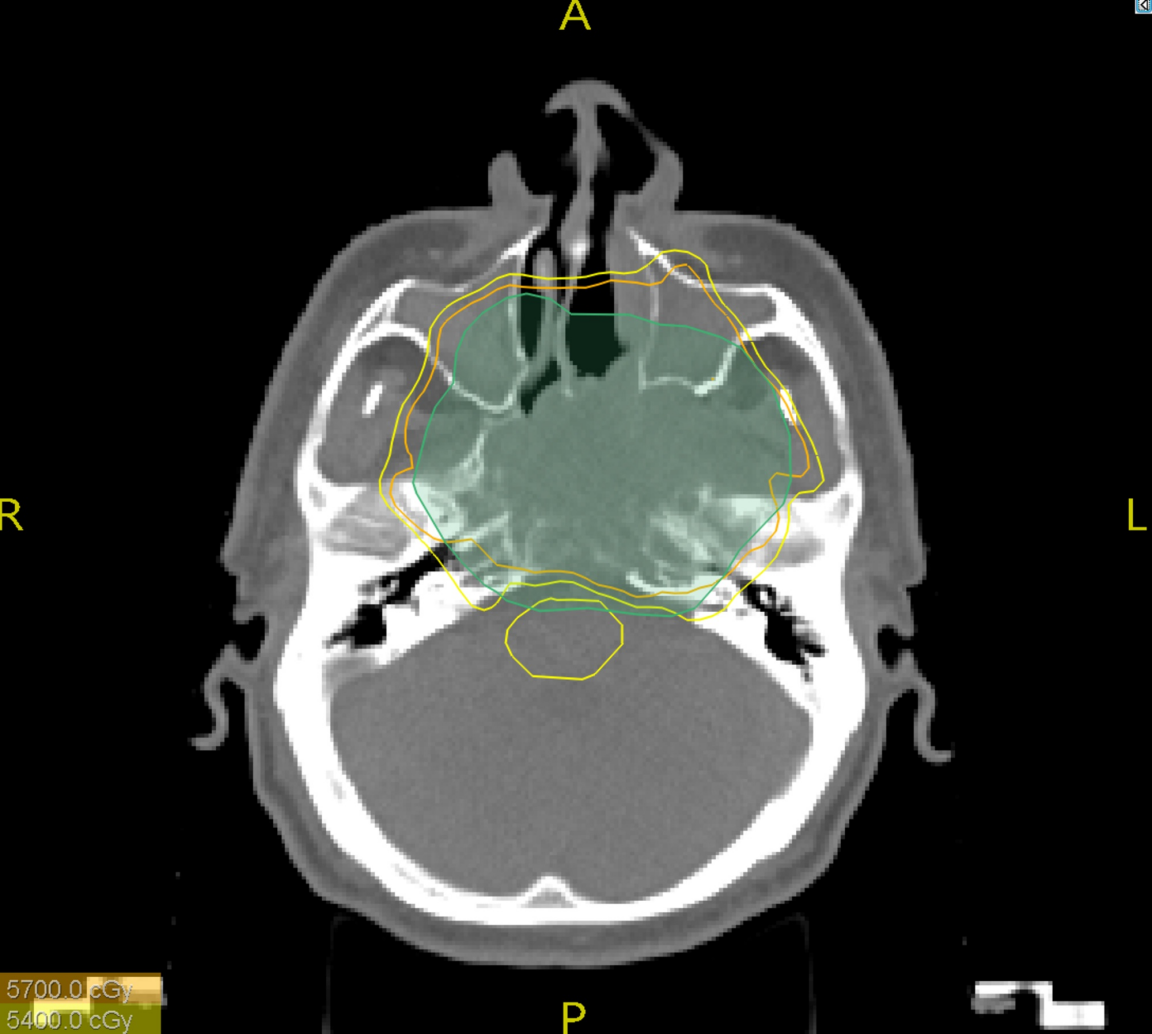
Axial image of a representative cut from the intensity-modulated radiation therapy plan showing adequate isodose lines coverage of the planning target volume. Green: planning target volume (PTV) Yellow: 90% isodose line Orange: 95% isodose line

The patient was seen by a head and neck surgeon who considered that the tumor was unresectable. The patient was treated with definitive intensity-modulated radiation therapy (IMRT). He received a total dose of 60 Gy in 30 fractions (Figure 3). During radiation treatment, the patient developed a grade 1 dermatitis and partial alopecia. No grade 2 or higher toxicities were reported.

**Figure 4:**
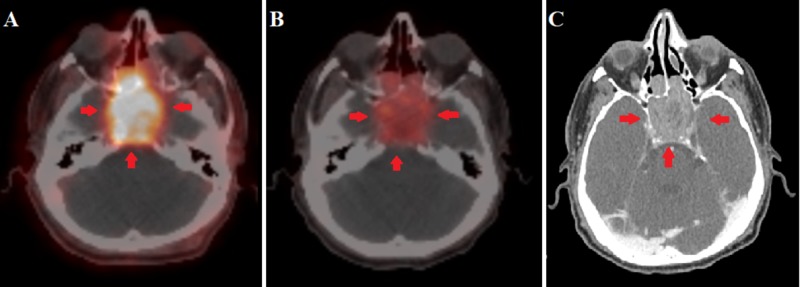
Gallium PET/CT scan (A, B); CT scan with contrast (C) (A) Baseline gallium positron emission tomography/computed tomography (PET/CT) scan showing the large hypermetabolic sphenoid sinus tumor; (B) PET-CT scan three months after intensity-modulated radiation therapy (IMRT) showing a significant decrease in metabolic activity; (C) CT scan at the last follow-up three years after diagnosis showing stable disease.

The patient had a follow-up galium PET/CT scan (three months after completion of IMRT) that showed a significant decrease in metabolic activity. The size was stable, and there was evidence of central necrosis (Figure 4A-4B). During his last follow-up, three years after diagnosis, a CT scan of the neck, chest, abdomen, and pelvis showed a stable tumor and no evidence of metastatic disease (Figure 4C)

## Discussion

Head and neck tumors represent around 3% of all cancers in the United States [5]. Squamous cell carcinoma accounts for the vast majority of cases. We have previously reported a case of locally advanced sinonasal basaloid squamous cell carcinoma, a rare tumor, that was treated aggressively with surgical resection and adjuvant IMRT radiation with good local control [6]. As for NETs, they comprise only a small percentage of the head and neck cases reported [5]. The most common site for head and neck NETs is the larynx, followed by salivary glands, whereas paranasal NETs account for only 5% of NET cases. The World Health Organization (WHO) 2017 classification divided NET based on tumor differentiation into typical carcinoid (well-differentiated neuroendocrine carcinoma), atypical carcinoid (moderately differentiated neuroendocrine carcinoma), and poorly differentiated neuroendocrine carcinoma (small and large cell subtype) [7]. In a review of the Surveillance, Epidemiology and End Results (SEER) database between 1973 and 2011, only 56 cases of the carcinoid type out of 1,346 head and neck NETs identified were reported [1].

The clinical and histological heterogeneity of NETs makes it a challenge for the pathologists to diagnose and the oncologists to treat. Well-differentiated NET (carcinoid) produces abundant secretory granules with intense immunoexpression of neuroendocrine markers, such as Syn, CgA, CD56, and cytokeratin AE1/AE3. These are characteristically arranged in a well-developed “organoid” or neuroendocrine shape with nesting, trabecular, or gyriform/serpentine growth patterns. Both the mitotic rate and the presence of necrosis represent criteria used to distinguish between typical and atypical carcinoids. Typical carcinoids are characterized by a low mitotic rate (< 2 mitoses per 2 mm² 10 high-power field (HPF)) and the absence of necrosis, whereas atypical carcinoids show a moderate mitotic rate (2 - 10 mitosis per 2 mm² 10 HPF) and/or the presence of necrosis. Immunostaining to S-100 is typically negative for NETs, and Ki67 index is usually low (< 3%) in carcinoid tumors [8]. Since the cells in our patient were uniformly round, had a low mitotic rate, and stained positive for CgA, CD65, Syn, and had an absence of S-100 immunostaining with a low Ki67 (< 1%), the pathological diagnosis was consistent with typical carcinoid.

To date, the treatment of head and neck NETs remains challenging and controversial, with surgery being the mainstay treatment. This has been illustrated in previously reported cases for patients with typical carcinoid tumors arising from the nasal cavity or paranasal sinuses [9-12]. However, due to the presence of extensive disease at initial presentation, surgical resection may not be an option in many cases similar to our patient. The role of radiotherapy in head and neck carcinoid tumors is limited. In other common tumor sites (for example, the lungs), radiotherapy has been used as adjuvant therapy or for unresectable tumors [13]. In a recent meta-analysis by van der Laan et al. on 701 cases of sinonasal NET, surgery was the cornerstone of treatment irrespective of tissue diagnosis [14]. Four patients in the meta-analysis had no histologic distinction of tumor differentiation (well or moderate) and they were treated with definitive radiotherapy alone. They had a similar prognosis to those patients who were treated surgically. The authors also found no benefit from chemotherapy for the treatment of such tumors [14]. Our patient was treated with definitive radiotherapy due to tumor unresectability and achieved good local control over a period of three years with close imaging and clinical follow-up.

## Conclusions

NETs represent a rare entity of head and neck cancer. We report a rare case of non-operable typical carcinoid tumor arising from the sphenoid sinus and achieved local control with IMRT alone. This treatment approach in patients with unresectable typical carcinoid of the head and neck warrants further investigation.
